# Bottom-trawling along submarine canyons impacts deep sedimentary regimes

**DOI:** 10.1038/srep43332

**Published:** 2017-02-24

**Authors:** Sarah Paradis, Pere Puig, Pere Masqué, Xènia Juan-Díaz, Jacobo Martín, Albert Palanques

**Affiliations:** 1 Departament de Física and Institut de Ciència i Tecnologia Ambientals, Universitat Autònoma de Barcelona, Bellaterra, 08193, Spain; 2 Department of Marine Geosciences, Marine Sciences Institute, Consejo Superior de Investigaciones Científicas, Barcelona, 08003, Spain; 3 School of Natural Sciences, Centre for Marine Ecosystems Research, Edith Cowan University, Joondalup, WA 6027, Australia; 4 Oceans Institute & School of Physics, The University of Western Australia, 35 Stirling Highway, Crawley, WA 6009, Australia; 5 Centro Austral de Investigaciones Científicas (CADIC-CONICET), Bernardo Houssay 200, V9410CAB Ushuaia, Tierra del Fuego, Argentina

## Abstract

Many studies highlight that fish trawling activities cause seafloor erosion, but the assessment of the remobilization of surface sediments and its relocation is still not well documented. These impacts were examined along the flanks and axes of three headless submarine canyons incised on the Barcelona continental margin, where trawling fleets have been operating for decades. Trawled grounds along canyon flanks presented eroded and highly reworked surface sediments resulting from the passage of heavy trawling gear. Sedimentation rates on the upper canyon axes tripled and quadrupled its natural (i.e. pre-industrialization) values after a substantial increase in total horsepower of the operating trawling fleets between 1960 s and 1970 s. These impacts affected the upper canyon reaches next to fishing grounds, where sediment resuspended by trawling can be transported towards the canyon axes. This study highlights that bottom trawling has the capacity to alter natural sedimentary environments by promoting sediment-starved canyon flanks, and by enhancing sedimentation rates along the contiguous axes, independently of canyons’ morphology. Considering the global mechanisation and offshore expansion of bottom trawling fisheries since the mid-20^th^ century, these sedimentary alterations may occur in many trawled canyons worldwide, with further ecological impacts on the trophic status of these non-resilient benthic communities.

There is a growing perception based on scientific evidence that we are currently entering a new epoch, the Anthropocene, where human activity has become the main driver of global environmental change^1^,^2^,^3^. Although most of these changes are evident on land^4^,^5^,^6^, even the most remote marine environments are not exempt of human impact^7^. Among the anthropogenic activities impacting the deep seafloor, commercial fish trawling is considered to be the most harmful due to its widespread geographical presence, recurrence, and intensity^8^,^9^.

Scraping and ploughing of the sea-floor are the most direct impacts of bottom trawling, whose heavy otter doors can leave behind significant furrows^10^ and lead to the formation of high turbid plumes of resuspended sediments that can produce thick and persistent bottom and intermediate nepheloid layers^11^,^12^,^13^,^14^. However, most of these impacts have been documented in shallow environments (less than 100 m in depth), where trawling disturbance usually coexist with natural processes (i.e. storms, tides, waves) that can also resuspend comparable amounts of sediment and thus overcome the effects of trawling^14^,^15^. However, traditional fishing grounds have been shifting to deeper habitats over the last 50 years^16^, requiring bigger trawling gears and larger vessels to haul them^17^. Since the deep sea-floor is usually less affected by most natural high-energy physical perturbations than shallow environments, it is expected that the resulting impacts of trawling activities are intensified, leading to high vulnerability and slow recovery rates in both the physical medium and the ecosystem it harbors^18^,^19^.

Bottom trawling fishing grounds are common in the vicinities of submarine canyons^20^,^21^,^22^, as they are ecological hotpots^23^,^24^ and important nursery areas for commercial species^25^,^26^. The impacts that bottom trawling generates in these environments have been thoroughly documented in La Fonera Canyon (also known as Palamós Canyon), on the Catalan margin off the NW Mediterranean. Subsequent studies have proven that dragging the heavy otter-trawls along the canyon’s flank has the capacity to remobilize large amounts of sediment, eroding fishing grounds and altering the complex geomorphology of canyon flanks^20^,^27^. Sediment resuspended in the wake of trawling gear is then intercepted by the canyon’s morphology and channelled downcanyon as diluted sediment gravity flows^28^,^29^, leading to deposition along the canyon floor as they slow down and lose their sediment load. As a consequence, sedimentation rates in some areas of the canyon axis have increased following the industrialization of the trawling fleet, altering the biological community of this deep environment^30^,^31^.

The Catalan margin is incised by many submarine canyons which hold important bottom trawling fishing grounds in its heads, rims, and flanks (Fig. 1), targeting the blue and red deep-sea shrimp, *Aristeus antennatus*, whose life-cycle is closely related to these seafloor morphological features^32^,^33^. Canyons in this passive margin act as sediment depocenters of particulate matter directly delivered by rivers or resuspended and advected off-shelf during storms and dense shelf water cascading events^34^,^35^, which is transported along the NW Mediterranean margin by the geostrophic Northern current^36^,^37^. The capacity of submarine canyons to intercept these suspended particles depends mostly on its incision length into the continental shelf and its distance to the shoreline^35^. This results in high particle fluxes in shelf-incised canyons such as Cap de Creus^38^, Palamós^39^ and Blanes^40^ canyons, which usually deflect the greatest sediment load originated by dense shelf water cascading and major storms events^41^. Moreover, given the micro-tidal characteristic of the enclosed Mediterranean Sea, internal waves within submarine canyons are close to the inertial frequency and the associated currents are weak, rendering this process relatively insignificant with respect to the local sediment resuspension mechanisms along these canyons^39^,^40^,^42^,^43^.

Given the ubiquitous occurrence of bottom trawling along the Catalan margin and the evolution of the corresponding trawling fleet^20^,^44^, similar alterations of the natural sedimentary environments observed in La Fonera Canyon might be present along other submarine canyons. In this study, the impacts of bottom fish-trawling are assessed in three headless submarine canyons, which hold important fishing grounds: Arenys, Besòs, and Morràs Canyons. These submarine canyons are non-incised in the continental shelf, and are presumably less affected by the natural off-shelf transport processes prevailing in the Catalan margin. The flanks of Morràs and Besòs canyons are assigned to the Barcelona fishing fleet, where trawling grounds are limited to 900 m in depth, whereas the flanks of the Arenys Canyon and the eastern flank of the Besòs Canyon are designated to the Arenys trawling fleet, where trawling rarely exceed 800 m in depth (Fig. 1).

The degree of erosion and changes in sediment accumulation rates were studied using the natural radioisotope ^210^Pb, whose half-life of 22.3 years allows the quantification of sedimentation rates over the last century^45^. Concentration profiles of the artificial radionuclide ^137^Cs were also obtained to corroborate ^210^Pb-derived sedimentation rates.

## Results

Results of the parameters analysed for all sediment cores are presented separately for the three studied canyons. Table 1 provides information on the location and sampling depth of all sediment cores and summarizes the main parameters derived from radionuclide analyses, while Figs 2 show the concentration profiles of excess ^210^Pb and ^137^Cs (when applicable). Supported ^210^Pb concentrations obtained either from complete decay of excess ^210^Pb in depth or from the quantification of ^226^Ra through gamma analyses were similar, ranging between 30 and 36 Bq·kg^−1^, and comparable to concentrations obtained in other studies from the Catalan margin^30^,^46^. Grain size and dry bulk density profiles of sediment cores are given in Supplementary Figs S1–S3.

### Morràs Canyon

#### Canyon flank

Sediment cores retrieved along the northern canyon flank of this submarine canyon at ~700 m water-depth, where trawling takes place, have similar grain size (73% and 25% of silt and clay) and very low sand content (Supplementary Fig. S1). In both cores, slight sediment coarsening is present in the upper layers, caused by an increasing fraction of sand and reduction of clay. Dry bulk densities of both sediment cores were similar in the surface layers (~0.6 g·cm^−3^). In Morràs 1, dry bulk density increased steadily down-core to ~0.8 g·cm^−3^ at 25 cm, where sediment reached maximum consolidation and remained constant with depth, while in Morràs 2 it rapidly reached ~0.8 g·cm^−3^ in the upper 5 cm and remained constant below.

The concentrations of excess ^210^Pb in the upper 10 cm of Morràs 1 were constant (averaging 196 ± 17 Bq·kg^−1^) and subsequently decreased until reaching the excess horizon at 25 cm, with a total inventory of 19600 ± 600 Bq·m^−2^ (Table 1; Fig. 2). A mass accumulation rate of 0.150 ± 0.011 g·cm^−2^·y^−1^ (0.207 ± 0.016 cm·y^−1^) was estimated from the excess ^210^Pb concentration profile below the upper mixed layer. In Morràs 2, superficial sediments presented low excess ^210^Pb concentrations (55 ± 10 Bq·kg^−1^) that rapidly decreased to a constant 8 ± 2 Bq·kg^−1^ excess ^210^Pb concentration at 3–10 cm, before reaching the excess ^210^Pb horizon (Fig. 2). The excess ^210^Pb inventory was of 900 ± 100 Bq·m^−2^, an order of magnitude lower than in Morràs 1. Sediment accumulation rates could not be calculated for Morràs 2.

#### Canyon axis

Grain size distributions of sediment cores retrieved from the Morràs canyon axis at ~800 m depth (Morràs 3) and ~1000 m depth (Morràs 4) were similar to those in the canyon flank, with predominant silt (74%) and clay (24%) fractions and minor presence of sand (<0.5%) (Supplementary Fig. S1). Dry bulk density profiles of both cores were similar, with low values of ~0.4 g·cm^−3^ at the surface that increased in the upper 10 cm to ~0.7 g·cm^−3^, followed by a steady increase in depth reaching similar maximum consolidation (~0.8 g·cm^−3^) as the sediment cores from the flanks (Supplementary Fig. S1).

The ^210^Pb concentration profile of Morràs 3 shows two different decreasing gradients in depth, with an average mass accumulation rate of 0.090 ± 0.005 g·cm^−2^·y^−1^ (0.128 ± 0.007 cm·y^−1^) in the lower sections overlain by a higher sediment mass accumulation rate of 0.184 ± 0.009 g·cm^−2^·y^−1^ (0.322 ± 0.016 cm·y^−1^) (Fig. 2). Although the change of ^210^Pb concentration in the upper 10 cm could have been confounded by mixing associated to bioturbation and physical reworking of sediments, the ^137^Cs time-markers support an almost tripling of the sedimentation rate in the early 1970 s: the 1986 Chernobyl accident at 6–7 cm, and the 1963 maximum fallout at 11–12 cm (Fig. 2). The gradually-decreasing base of the ^137^Cs profile imply that this radioisotope has presented a certain downward mobility^46^,^47^ so the 1950 s time-marker could not be identified.

On the other hand, there is no evidence of a change in sedimentation rate in Morràs 4, as shown by the single decreasing excess ^210^Pb slope, below a 4 cm surface mixed layer, that corresponds to a sediment accumulation rate of 0.129 ± 0.005 g·cm^−2^·y^−1^ (0.200 ± 0.007 cm·y^−1^). This core did not present clear ^137^Cs maxima, although a relative increase at 8 cm could correspond to the 1986 Chernobyl accident. Nevertheless, the sharp base of the ^137^Cs profile, attributable to the early 1950 s, validates the ^210^Pb-derived sedimentation rates.

### Besòs Canyon

In the Besòs Canyon, sediment characteristics of the core collected at ~800 m depth (Besòs 1) differed slightly from the other deeper sediment cores collected at ~1200 m depth (Besòs 2) and ~1500 m depth (Besòs 3). In these latter cores, the grain size fractions consisted mainly of silt (76 ± 4% and 66 ± 3%) and clay (23 ± 4% and 33 ± 3%) for Besòs 2 and 3 respectively, with a minor sand fraction in both cores (<0.5%). These two cores presented a slight coarsening in the upper layers from increased silt to clay proportion. In the case of Besòs 1, although silt (74 ± 3%) and clay (21 ± 2%) also predominated, higher proportions of coarser sediment were present: 5 ± 3% of sand along the core and 2–4% of gravel in some deep layers (43–44 cm) (Supplementary Fig. S2).

Besòs 2 and 3 also presented different dry bulk density profiles from Besòs 1. In Besòs 2 and 3, bulk density of surface unconsolidated sediment (<0.4 g·cm^−3^) rapidly increased in the first 10 cm to approximately 0.7 g·cm^−3^, from where it then gradually increased with depth to its maximum consolidation (~0.8 g·cm^−3^). In the case of Besòs 1, dry bulk density increased steadily throughout the whole profile from 0.6 g·cm^−3^ to almost 0.8 g·cm^−3^ in its bottom-most layer (Supplementary Fig. S2), which suggests a higher sedimentation in this site in comparison to sediment cores retrieved farther down-canyon.

The horizon of excess ^210^Pb profile of Besòs 1 was not reached despite the length of this core (55 cm), thus its inventory is estimated to be greater than 54 000 Bq·m^−2^. The concentration profile of excess ^210^Pb displays a surface mixed layer in the upper 4 cm, followed by two similarly-decreasing trends of ^210^Pb with equivalent sedimentation rates of 0.63 ± 0.06 g·cm^−2^·y^−1^ (0.98 ± 0.08 cm·y^−1^) and 0.61 ± 0.04 g·cm^−2^·y^−1^ (0.92 ± 0.06 cm·y^−1^), interrupted by a layer of constant ^210^Pb concentrations at 25–28 cm, that is interpreted to be the result of a rapid sedimentation pulse-event during the early 1990 s (Fig. 3). Below 40 cm, a gentler ^210^Pb slope reveals a lower sedimentation rate of 0.28 ± 0.03 g·cm^−2^·y^−1^ (0.39 ± 0.04 cm·y^−1^), indicating that recent sedimentation rates almost tripled over the early 1970 s (Fig. 3). These sediment accumulation rates, along with the pulse-event, were corroborated by the ^137^Cs concentration profile, whose base, dated in the early 1950 s, and the overhead concentration maximum, attributed to the 1986 Chernobyl accident, are preserved (Table 1; Fig. 3). The 1963 time-marker is not clearly detected in this core, but based on the shape of the ^137^Cs concentration profile it could correspond to 41 cm, which also concurs with the ^210^Pb-derived ages.

In Besòs 2, excess ^210^Pb concentrations decreased with depth along the core, with an intermediate constant concentration between 12 and 15 cm in depth (Fig. 3). Similar sedimentation rates were obtained below and above this layer, 0.118 ± 0.008 g·cm^−2^·y^−1^ (0.180 ± 0.012 cm·y^−1^) and 0.110 ± 0.002 g·cm^−2^·y^−1^ (0.205 ± 0.005 cm·y^−1^), respectively, interrupted by a rapid sedimentation pulse-event dated in the early 1960 s. The concentration profile of ^137^Cs clearly preserves the relative maxima corresponding to the Chernobyl accident in 1986, which agrees with the ^210^Pb-derived ages (Fig. 3). The 1963 time-marker is not easily identifiable, since it is affected by the presence of the pulse-event, although a concentration maximum at 14 cm may suggest its presence during this episode, which also concurs with ^210^Pb-derived ages. In depth, the 1950 s time-marker is not identified since the gradually-decreasing concentration of ^137^Cs at the base of this profile indicates some downward mobility of this radioisotope (Table 1; Fig. 3).

Farther down-canyon, the ^210^Pb concentration profile of Besòs 3 reveals a 5 cm surface mixed layer, overlaying a sedimentation rate of 0.057 ± 0.002 g·cm^−2^·y^−1^ (0.078 ± 0.003 cm·y^−1^). This sedimentation rate is corroborated by the ^137^Cs relative maximum at 5 cm, which is attributed to the 1963 peak fallout (Fig. 3). Considering the low sedimentation rate of this sediment core, ^137^Cs may have been subjected to minor mobility, displacing the base of ^137^Cs slightly downwards (Fig. 3). The 1986 Chernobyl time-marker is not preserved, as it would have been located in the surface mixed layer.

### Arenys Canyon

Sediment cores retrieved along the Arenys Canyon axis at ~1100, ~1400 and ~1650 m depth consist mostly of silt (74%) and clay (25%), with minor sand fraction (<0.5%). As observed in the other canyons, there is also a gradual coarsening of grain size in the upper layers caused by an increase in the contents of silt (Supplementary Fig. S3).

Dry bulk density in Arenys 1 steadily increased from ~0.4 g·cm^−3^ at the surface to ~0.9 g·cm^−3^ at 15 cm in depth, remaining constant down to the bottom of the core. For both Arenys 2 and 3, dry bulk density increased more rapidly, reaching ~0.8 g·cm^−3^ at about 10 cm (Supplementary Fig. S3).

The excess ^210^Pb concentration profile of Arenys 1 presents a thin surface mixed layer of 2 cm and, below, two distinct slopes attributable to two different sediment accumulation rates: 0.061 ± 0.004 g·cm^−2^·y^−1^ (0.078 ± 0.005 cm·y^−1^) that increased to 0.176 ± 0.009 g·cm^−2^·y^−1^ (0.297 ± 0.015 cm·y^−1^) in the early 1970 s. These sediment accumulation rates are confirmed by the three identified ^137^Cs time-markers (Fig. 4; Table 1). The higher sedimentation rate in the upper layers is also evident in the dry bulk density profiles, where sediment in these layers presented a lower compaction in depth compared to Arenys 2 and 3 (Supplementary Fig. S3).

For cores Arenys 2 and 3, excess ^210^Pb concentrations decrease following a single trend in each core, with comparable sediment accumulation rates: 0.066 ± 0.004 g·cm^−2^·y^−1^ (0.093 ± 0.006 cm·y^−1^) for Arenys 2 and 0.061 ± 0.002 g·cm^−2^·y^−1^ (0.091 ± 0.003 cm·y^−1^) for Arenys 3 (Fig. 4; Table 1).

### Evolution of the fishing fleet

The Barcelona trawling fleet experienced its greatest expansion during the 1950–1970 s, when total number of operating trawlers doubled (from 12 to 24 active trawlers) with the construction of new and more powerful vessels (Fig. 5a), leading to a 5-fold increase in registered total horsepower (from 1050 to 5500 HP). Total horsepower of this fishing fleet peaked during the 1990 s, after which it decreased by 30% (from ~7300 HP to ~5100 HP) along a noteworthy dismantling of 29 trawlers, replaced by 14 new trawlers. Despite this net decrease, the average horsepower still increased by 50% over this period (Fig. 5a).

The growth of the Arenys trawling fleet was delayed by a decade in comparison to Barcelona and it occurred over the 1960–1980 s, also doubling (from 8 to 17) and leading to an almost 6-fold increase in registered total horsepower (from 750 to 4350 HP). These increases were accompanied by a three-fold increase in average horsepower over this period (Fig. 5b). As with the Barcelona fishing fleet, total horsepower peaked over the 1990 s and then almost halved over the following decades (from ~5500 HP to ~3100 HP), with 15 trawlers dismantled and replaced by 9 new ones. Despite this overall decrease, the average horsepower remained constant during this period (Fig. 5b).

## Discussion

**Figure 1:**
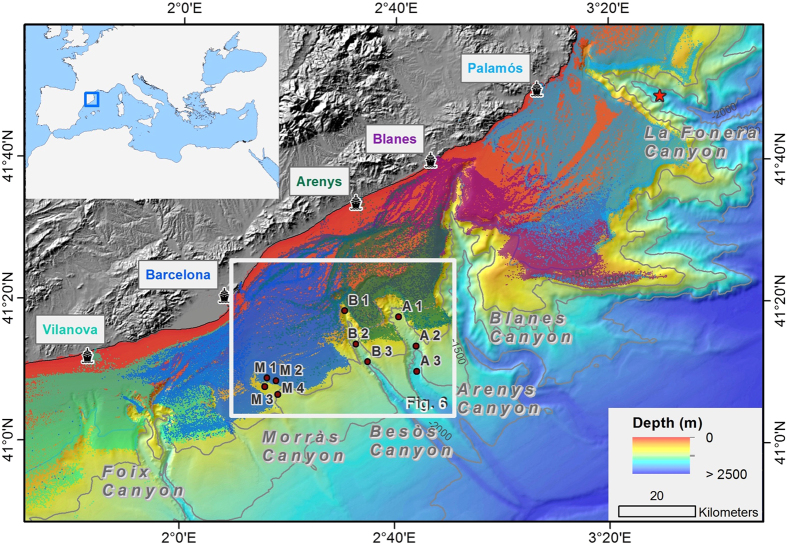
Bathymetric map of the Catalan margin showing the incised submarine canyons, and the extension of bottom-trawling fishing grounds. Colored dots indicate VMS positioning over 2005–2011 of bottom trawlers of Vilanova, Barcelona, Arenys, Blanes, and Palamós ports (see color code). The studied canyons and locations of sediment cores retrieved are indicated by red circles and labelled according to their corresponding canyon name (A: Arenys, B: Besòs, M: Morràs). The red star shown within La Fonera Canyon axis indicates the location of sediment cores analysed in ref. 31 and 32. This map was generated using ArcGIS 10.3 (http://desktop.arcgis.com/en/arcmap/10.3/main/get-started/whats-new-in-arcgis.htm). Bathymetric information was obtained from a north-western Mediterranean Digital Elevation Model^66^.

Several impacts caused by bottom trawling are evident in the sediment cores collected in the Barcelona continental margin (Fig. 1). Regarding the alterations observed on canyon flanks, Morràs 1, retrieved on fishing grounds along the Morràs Canyon flank at ~700 m depth, exhibits concentrations of excess ^210^Pb in the upper 10 cm that are constant and lower than expected considering the water depth of this core^46^. This might result from the piling of overworked sediments by the passage of trawling gears, as evidenced on ROV footage of trawled flanks of La Fonera Canyon^20^. Interestingly, the depth of reworked sediments in this core concurs with estimated penetration depths of otter trawls in muddy sediments^10^,^48^, which upholds this hypothesis.

**Figure 2:**
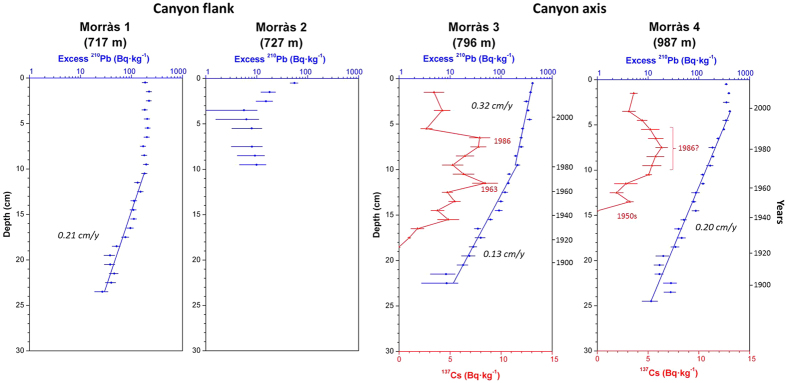
Concentration profiles (^210^Pb in blue and ^137^Cs in red when applicable) of sediment cores retrieved on fishing grounds of the Morràs Canyon flank (1 and 2), and on its axis (3 and 4).

For Morràs 2, also retrieved on fishing grounds at ~700 m depth, the surface excess ^210^Pb concentrations were an order of magnitude lower than in the adjacent core Morràs 1, while the horizon of excess ^210^Pb and the maximum consolidation of sediment were reached at barely 10 cm (Fig. 2, Supplementary Fig. S1), leading to a substantially lower inventory of 900 ± 100 Bq·m^−2^ in comparison to 19 600 ± 600 Bq·m^−2^ calculated for Morràs 1 (Table 1). All this suggests that a significant amount of sediment was removed by the passage of heavy trawling gears, which can be estimated as almost a century-worth of sediment deposited in this area. The constant excess ^210^Pb concentrations observed between 3 and 10 cm can be related to heavily reworked sediments homogenized by the passage of the trawling gear, further highlighting the capacity of otter trawls to erode and homogenize the seafloor.

**Figure 3:**
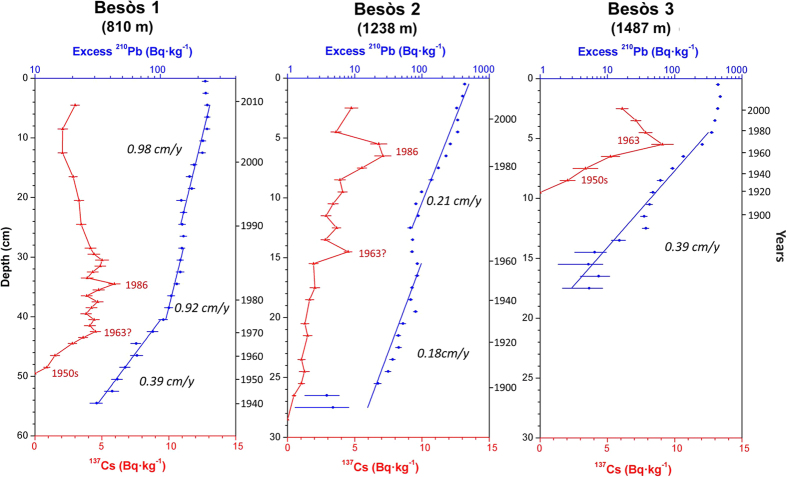
Concentration profiles (^210^Pb in blue and ^137^Cs in red when applicable) of sediment cores retrieved in the Besòs Canyon axis. Note the change of scale for the depth and excess ^210^Pb axes of Besòs 1.

**Figure 4:**
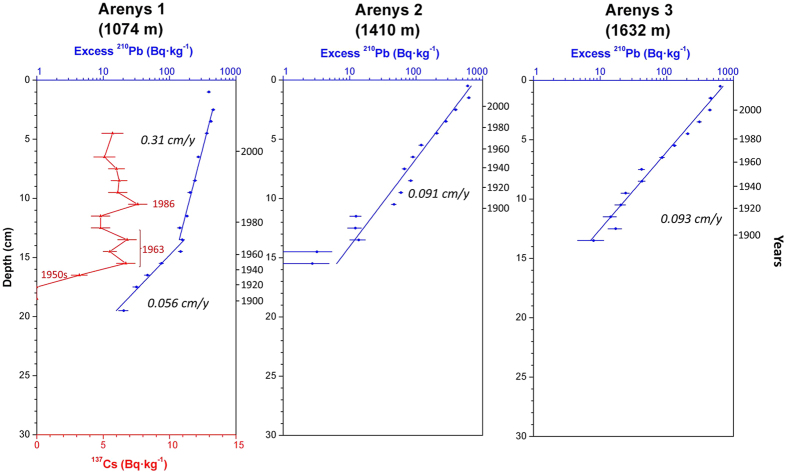
Concentration profiles (^210^Pb in blue and ^137^Cs in red when applicable) of sediment cores retrieved in the Arenys Canyon axis.

**Figure 5:**
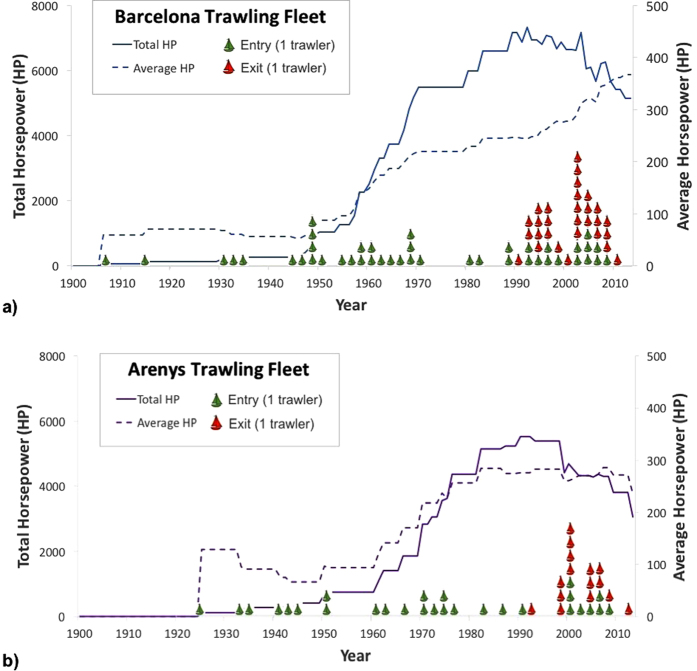
Evolution of the Barcelona (**a**) and Arenys (**b**) trawling fleets in terms of total horsepower, average horsepower, and entry and removal of vessels. Number of bottom trawlers entering and exiting the active fleet are shown over a two-year interval.

**Figure 6:**
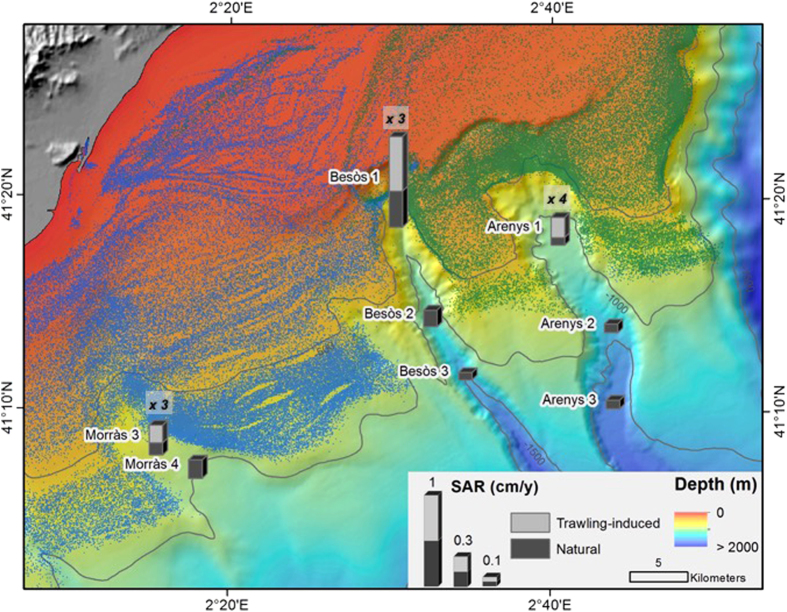
Trawl-induced and natural sediment accumulation rates in the studied submarine canyons. Stacked bars are divided by the natural sedimentation rate and the overall enhancement of sedimentation rates as a consequence of trawling modernization (trawling-induced) sediment resuspension associated to this activity. Numbers next to each sediment core affected by trawling activities indicate the increase factor of sedimentation associated to trawling activities after the industrialization of the trawling fleet (1960 s–1970 s). This map was generated using ArcGIS 10.3 (http://desktop.arcgis.com/en/arcmap/10.3/main/get-started/whats-new-in-arcgis.htm). Bathymetric information was obtained from a north-western Mediterranean Digital Elevation Model^66^.

Concerning the cores taken within canyon axes, increases in sedimentation rates were observed in all sediment cores retrieved in the upper canyon region (i.e., Morràs 3, Besòs 1 and Arenys 1), starting synchronously in the late 1960 s - early 1970 s (Figs 2,3,4 and 6). These increases are unlikely attributable to the arrival of allochtonous sediments transported by natural processes, since there is no evidence in the study area of an intensification of storms or dense shelf water cascading events during the last decades. Moreover, the sediment yields from rivers discharging in the area have been drastically reduced by 75 to 90% since the early 1960 s, mostly due to river damming^49^, limiting the riverine sediment inputs to the margin. Therefore, such increases of sedimentation rates within submarine canyons have been interpreted here as being associated to the sediment resuspension caused by trawling activities since their industrialization (Fig. 5) and to a greater sediment input carried by the regional Northern current along the upper and mid-slope depths due to this activity. The excess ^210^Pb concentration profiles in these cores indicate that this enhanced sedimentation is a continuous process, and cannot be the result of localized sediment mass-transport events. If so, they would have generated non-steady ^210^Pb profiles or levels of constant excess ^210^Pb concentrations, and possibly a distinct signature in the grain size fraction associated to these events. In this regard, only the shallower cores collected in the Besòs Canyon exhibited sediment layers that could be interpreted as generated by rapid sedimentation events, most likely derived from slope instabilities and mass failures from the canyon flanks (see discussion below).

Within the Morràs canyon, only Morràs 3, collected at ~700 m depth and located within the trawling depths of the Barcelona fleet, presents a significant (threefold) increase of sediment accumulation rate during the early 1970 s. Despite the closeness of Morràs 4 to trawling grounds, it remained unaffected. This sediment core was collected at ~1000 m depth, deeper in relation to the trawlers’ 900 m maximum depth extension, thus being out of reach of the advection of the enhanced sediment input that could ultimately alter its sedimentation rates (Figs 2 and 6).

In the narrow Besòs canyon axis, only Besòs 1 located in the upper canyon ~800 m depth and down-current of trawling grounds showed a threefold increase in sedimentation rates during the early 1970 s (Fig. 3). Both this sediment core and Besòs 2, located at ~1200 m depth, presented sediment pulse-events, evidenced by layers of constant ^210^Pb concentrations along with constant grain size throughout the cores (Fig. 3 and Supplementary Fig. S2). As previously mentioned, these events may result from submarine canyon slope failures, which are usually triggered by external factors that can destabilize sediment along the canyon flanks^50^. These pulse-events occurred in coincidence with maximum total horsepower in 1990 s for Besòs 1 and the period of greatest modernisation of the fishing fleet in the early 1960 s for Besòs 2 (Figs 3 and 5), suggesting that this rapid accumulation may also have been set off by trawling. After these events, sediment rates returned to those enhanced by trawling in Besòs 1 and to the rates not affected by trawling in Besòs 2, since it is out of reach of the enhanced input of trawled sediment (Figs 3 and 6), as is the case of Besòs 3, located further down-canyon at ~1500 m depth.

Finally, the Arenys canyon axis presented a fourfold increase in sediment accumulation rates in the early 1970 s on the shallower coring site next to trawling grounds, whereas in deeper canyon areas, far away of the trawled flanks, sedimentation rates remained unaffected (Figs 4 and 6).

To our knowledge, the evident capacity of trawling activities to resuspend and erode sediments on the canyon flanks^20^,^27^, ultimately increasing sediment accumulation rates in submarine canyon environments due to the modernisation of the local trawling fleet, has only been reported in one coring site at La Fonera Canyon axis, around 1700 m depth^30^,^31^ (Fig. 1). A similar upgrade occurred in the Barcelona and Arenys trawling fleets in terms of number of operating vessels and their total and average horsepower (Fig. 5). Over the late 1960 s and early 1970 s, new trawlers became highly technicized, accompanied by the expansion of fishing grounds to deeper depths^16^, which require greater engine power to haul the heavier gears^17^, thus increasing their capacity to resuspend sediment. This resulted in an enhanced input of sediment over the slope region that can be transported by the regional Northern current, increasing sedimentation rates over this period in the upper reaches of the Morràs, Besòs, and Arenys canyons (Fig. 6).

Our observations provide new evidences of the capacity of trawling activities to modify natural sedimentary processes in continental slope environments. Trawling-induced alterations of sedimentation processes within the studied submarine canyons is limited to their upper reaches, where trawling takes place in the contiguous flanks, tripling and quadrupling sedimentation rates on their axes since the period of greatest expansion of the operating trawling fleet (Fig. 5). These results highlight that the effects of trawling in canyon sedimentary regimes are confined to a specific area downslope from trawling grounds, leaving farther and deeper areas in the canyon unaffected (Fig. 6). Our study suggests that submarine canyons impacted by trawling, including those non-incised in the continental shelf, can have altered sedimentary regimes along their flanks and axes, creating anthropogenic sedimentary depocenters.

Indeed, the evolution of the bottom trawling fleets of the ports of Barcelona and Arenys are not only in accordance with those of the port of Palamós^30^, but also with all bottom trawler fisheries operating in the Mediterranean Sea, which have shifted their target species to deep-water crustaceans over this same period^51^.The most relevant bottom trawling European fisheries in the Mediterranean Sea (Italy, Spain, and Greece) augmented substantially between the 1960 s and 1980 s (Supplementary Fig. S4). This modernization is especially evident for the Italian trawling fleet, which experienced an order of magnitude increase of its fishing fleet and total horsepower, while Spain and Greece tripled the number of operating vessels and total horsepower during this period. Taking this into account and the extension of trawling activities along the canyoned Mediterranean margin^52^, it is likely that they have all experienced perturbations in their sedimentary regime: transformation of canyon flanks into sediment-starved areas and enhancement of sedimentation rates within the proximal canyon axis. Considering the global expansion of bottom trawling fisheries^20^, similar impacts might be occurring in other submarine canyons at a global scale.

Until recently, the awareness of bottom trawling impacts has been mostly restricted to fishing grounds^10^,^11^,^12^, but trawling-derived impacts are not limited to this area. The enhanced sedimentation rates evidenced in La Fonera^30^,^31^, and now in Arenys, Besòs, and Morràs canyons, which have received three to four times more sediment since the 1970 s, can lead to several ecological implications. On one hand, the arrival of such substantial volume of sediment over several decades to these low resilient environments can smother benthic organisms and alter their communities^31^,^48^,^53^,^54^. Moreover, submarine canyons are important habitats for cold water corals, but only occupy areas where hydrodynamics prevent high sedimentation rates^55^. Recent ROV footage of La Fonera Canyon have identified scarce cold water coral colonies in areas with presumably high sediment input induced by trawling along the contiguous flank, suggesting that sediment concentrations have surpassed these corals’ threshold^56^. On the other hand, sediment that is being accumulated come from nutrient-depleted sediment found on trawling grounds^57^,^58^, which may also affect the trophic status of benthic communities in these deep environments that rely on the arrival of sediment rich in organic matter. These extended impacts of trawling activities within canyon axes should be considered when establishing deep bottom-trawling regulations.

## Methods

### Sampling

A total of 10 sediment cores were obtained during several oceanographic campaigns aboard the R/V *García del Cid*: two along the flanks of the Morràs Canyon and two more in its axis, and three sediment cores along the axes of Besòs and Arenys submarine canyons (Table 1; Fig. 1). The sediment cores were all retrieved using a KC Denmark A/X 6-tube (inner diameter 9.4 cm) multicorer that can recover sediment cores of up to 60 cm in length. In every operation, the best-preserved core was chosen for analysis, based on a clear and undisturbed sediment-water interface that indicates minimal sediment disturbance during sampling. Sediment cores were then subsampled at 1 cm intervals and each section was kept frozen in sealed plastic bags until analysis.

### Analytical Procedures

Frozen samples were first weighed and then lyophilized for approximately 30 hours using a Laboratory Freeze Dryer at −50 °C and 0.005 mbar or lower. Dry weights were recorded and dry bulk density was determined by dividing the net dry weight corrected for salt content by the sample volume, the latter estimated assuming a seawater density of 1.025 g·cm^−3^ and an average grain density of 2.65 g·cm^−3^.

Grain size fraction was determined using a Horiba Partica LA-950V2 particle-size analyzer, with an accuracy of 0.6% and 0.1% precision. Prior to this analysis, 1–4 g of each sample were oxidized using 20% H_2_O_2_ for a week and then left overnight with a solution of P_2_O_7_^-^ to disaggregate the sediment particles.

Concentrations of ^210^Pb were determined through the analysis of the activity of its granddaughter ^210^Po by alpha-spectroscopy^59^, assuming secular equilibrium of both radionuclides at the time of analysis. Samples were traced using ^209^Po, followed by an acid digestion using an analytical microwave, and polonium isotopes were subsequently plated on silver discs. Alpha emissions were quantified using Passivated Implanted Planar Silicon (PIPS) detectors (CANBERRA, Mod. PD-450.18 A.M) with a MaestroTM data acquisition software. Supported ^210^Pb concentrations were determined by averaging total ^210^Pb concentrations at the base of each profile, verified with the ^226^Ra concentrations obtained by gamma-spectroscopy. This latter method was employed in cores where the horizon of excess ^210^Pb was not reached. Concentrations of ^226^Ra were determined through the emission peaks of its two decay products ^214^Pb (295 and 352 keV peaks) and ^214^Bi (609 keV peak) using a high-purity germanium detector (CANBERRA, mod. GCW3523) in calibrated geometries, sealed for 21 days to reach secular equilibrium with ^214^Pb and ^214^Bi. In some sediment cores, the concentration profiles of ^137^Cs were quantified using gamma-spectroscopy. Sediment accumulation rates were calculated based on accumulated dry mass to correct for sediment compaction in depth, applying the constant flux : constant sedimentation (CF:CS) model^60^. Given that biological and physical reworking of superficial sediments can alter the excess ^210^Pb concentration profiles, which can be confounded as being produced by greater sedimentation rates, the congruency of the ^210^Pb derived dates was corroborated with the ^137^Cs time-markers^61^. These time-markers are: i) the depth at which ^137^Cs is detected, dated on the early 1950 s, resulting from the first detonations of thermonuclear weapons and consequent significant emissions of artificial radioactivity to the atmosphere during these years^62^; ii) a relative concentration maxima corresponding to the time at which maximum deposition occurred prior to the cessation of nuclear atmospheric testing in 1963, and iii) a second relative concentration maxima derived from the emissions of the Chernobyl accident in 1986^47^,^63^.

### Ancillary Data

Positioning of vessels operating on the Barcelona continental margin was obtained from the Fishing Monitoring Centre of the Spanish General Secretariat of Maritime Fishing (SEGEMAR) as Vessel Monitoring System (VMS) data, a protocol established by the Common Fisheries Policy of the European Union^64^. Vessels equipped with VMS provide their position by Global Positioning System with an error margin of 100 m and transmit this information by Inmarsat-C to the Fishing Monitoring Centre in less than 10 min at 2-hour time intervals^65^. This database also holds information of the vessel such as its heading, speed, and registered harbor. The spatial distribution of large trawlers (boat length greater than 15 m) on the Barcelona continental margin for the period 2005–2011 is shown in Fig. 1.

Fishing grounds along the Arenys, Besòs, and Morràs canyons are trawled by vessels from the Port of Arenys and the Port of Barcelona, as confirmed from VMS data (Fig. 1). Additional information of trawlers from these ports was obtained from the Community Fishing Fleet Register^64^, an online European Union database that registers the full history of fishing vessels from a Member State along with their characteristics.

Information of bottom trawlers from other European countries with the most relevant bottom trawling fisheries in the Mediterranean Sea^51^ was also obtained from the Community Fishing Fleet Register. Full histories of bottom otter trawls of Spain (Mediterranean harbours), Italy and Greece were collected along with their operating ports.

## Additional Information

**How to cite this article**: Paradis, S. *et al*. Bottom-trawling along submarine canyons impacts deep sedimentary regimes. *Sci. Rep.* **7**, 43332; doi: 10.1038/srep43332 (2017).

**Publisher's note:** Springer Nature remains neutral with regard to jurisdictional claims in published maps and institutional affiliations.

## Supplementary Material

Supplementary Information

## Figures and Tables

**Table 1:** Sampling data of each core and the main parameters derived from radionuclide analysis of all sediment cores (MAR: Mass Accumulation Rate; SAR: Sediment Accumulation Rate).

Station	Sampling date	Location	Depth (m)	Supported ^210^Pb horizon (cm)	Surface excess ^210^Pb	Inventory	Accumulation Rates		
					(Bq·kg^−1^)	(Bq·m^−2^)	Layers (cm)	MAR (g·cm^−2^·y^−1^)	SAR (cm·y^−1^)
Morràs 1	13/10/2013	Canyon flank	717	25	180 ± 20	19 600 ± 600	10–25	0.150 ± 0.011	0.207 ± 0.016
Morràs 2	13/10/2013	Canyon flank	727	10	55 ± 10	900 ± 100	—	—	—
Morràs 3	13/10/2015	Canyon axis	796	23	410 ± 20	21 800 ± 500	0–11	0.184 ± 0.009	0.322 ± 0.016
							10–22	0.090 ± 0.005	0.128 ± 0.007
Morràs 4	13/10/2013	Canyon axis	987	26	350 ± 20	20 100 ± 500	3–25	0.129 ± 0.005	0.200 ± 0.007
Besòs 1	15/09/2014	Canyon axis	810	>55	230 ± 10	>54 000	4–25	0.63 ± 0.06	0.98 ± 0.08
							28–41	0.61 ± 0.04	0.92 ± 0.06
							40–54	0.28 ± 0.03	0.39 ± 0.04
Besòs 2	28/07/2012	Canyon axis	1238	28	430 ± 20	19 200 ± 200	0–12	0.110 ± 0.002	0.205 ± 0.005
							15–28	0.118 ± 0.006	0.180 ± 0.009
Besòs 3	29/07/2012	Canyon axis	1487	16	470 ± 20	14 000 ± 200	4–16	0.057 ± 0.002	0.078 ± 0.003
Arenys 1	28/07/2012	Canyon axis	1074	20	390 ± 20	20 500 ± 400	2–14	0.176 ± 0.009	0.297 ± 0.015
							13–20	0.061 ± 0.004	0.078 ± 0.005
Arenys 2	29/07/2012	Canyon axis	1410	16	600 ± 30	11 900 ± 200	0–16	0.066 ± 0.004	0.093 ± 0.006
Arenys 3	29/07/2012	Canyon axis	1632	15	630 ± 30	11 100 ± 300	0–14	0.061 ± 0.002	0.091 ± 0.003
