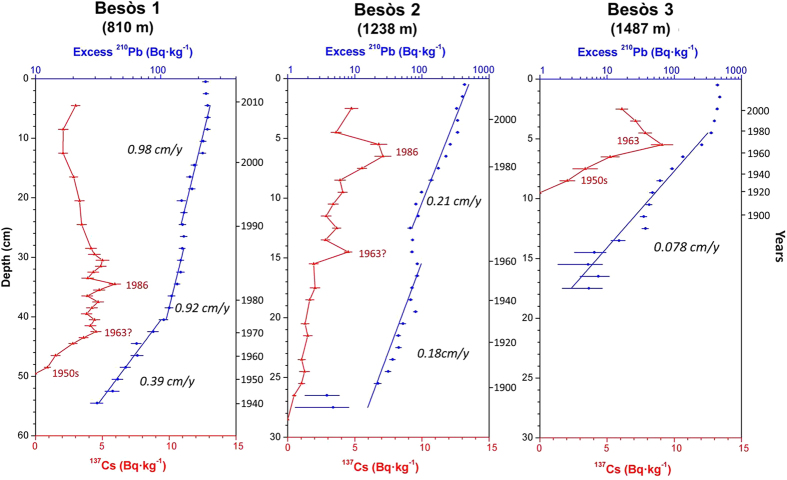# Corrigendum: Bottom-trawling along submarine canyons impacts deep sedimentary regimes

**DOI:** 10.1038/srep46903

**Published:** 2017-10-20

**Authors:** Sarah Paradis, Pere Puig, Pere Masqué, Xènia Juan-Díaz, Jacobo Martín, Albert Palanques

Scientific Reports 7: Article number: 43332; 10.1038/srep43332 published online: 02 24 2017; updated: 10 20 2017.

This Article contains an error in Figure 3, where the sedimentation rate from Besòs-3 ‘0.078 cm/y’ is incorrectly given as ‘0.39 cm/y’.

The correct Figure 3 appears below as Figure 1.

## Figures and Tables

**Figure 1:**