# Supplementary material for: Bottom-trawling along submarine canyons impacts deep sedimentary regimes
Source: Sci Rep. 2017 Feb 24;7:43332. doi: 10.1038/srep43332 (PMC5324136; doi:10.1038/srep43332)
Supplement: Supplementary Information [file srep43332-s1.pdf]

Supplementary Information

Bottom-trawling along submarine canyons impacts deep sedimentary regimes

Sarah Paradis[1,*], Pere Puig[2], Pere Masqué[1,3,4], Xènia Juan-Díaz[1], Jacobo Martín[5] and Albert Palanques[2]

[1] Departament de Física and Institut de Ciència i Tecnologia Ambientals, Universitat Autònoma de Barcelona, Bellaterra, 08193, Spain

[2] Department of Marine Geosciences, Marine Sciences Institute, Consejo Superior de Investigaciones Científicas, Barcelona, 08003, Spain

[3] School of Natural Sciences, Centre for Marine Ecosystems Research, Edith Cowan University, Joondalup, WA 6027, Australia

[4] The University of Western Australia Oceans Institute, University of Western Australia, 35 Stirling Highway, Crawley, WA 6009, Australia

[5] Centro Austral de Investigaciones Científicas (CADIC-CONICET), Bernardo Houssay 200, V9410CAB Ushuaia, Tierra del Fuego, Argentina

*Corresponding author: Sarah.Paradis@uab.cat

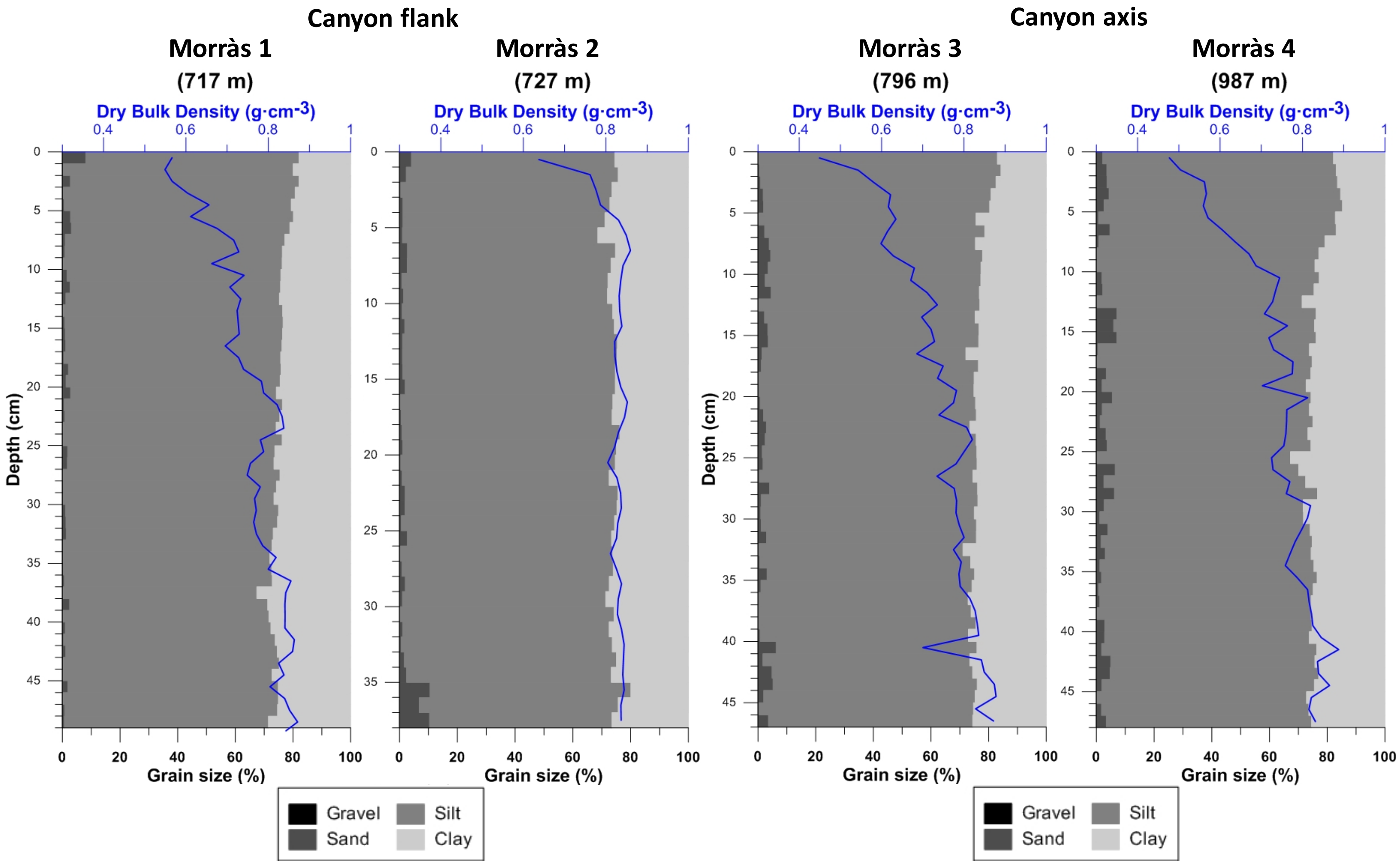

Supplementary Figure S1. Dry bulk density (blue lines) and grain size fraction (grey scales) profiles of sediment cores retrieved in the Morràs Canyon, in its flank and axis.

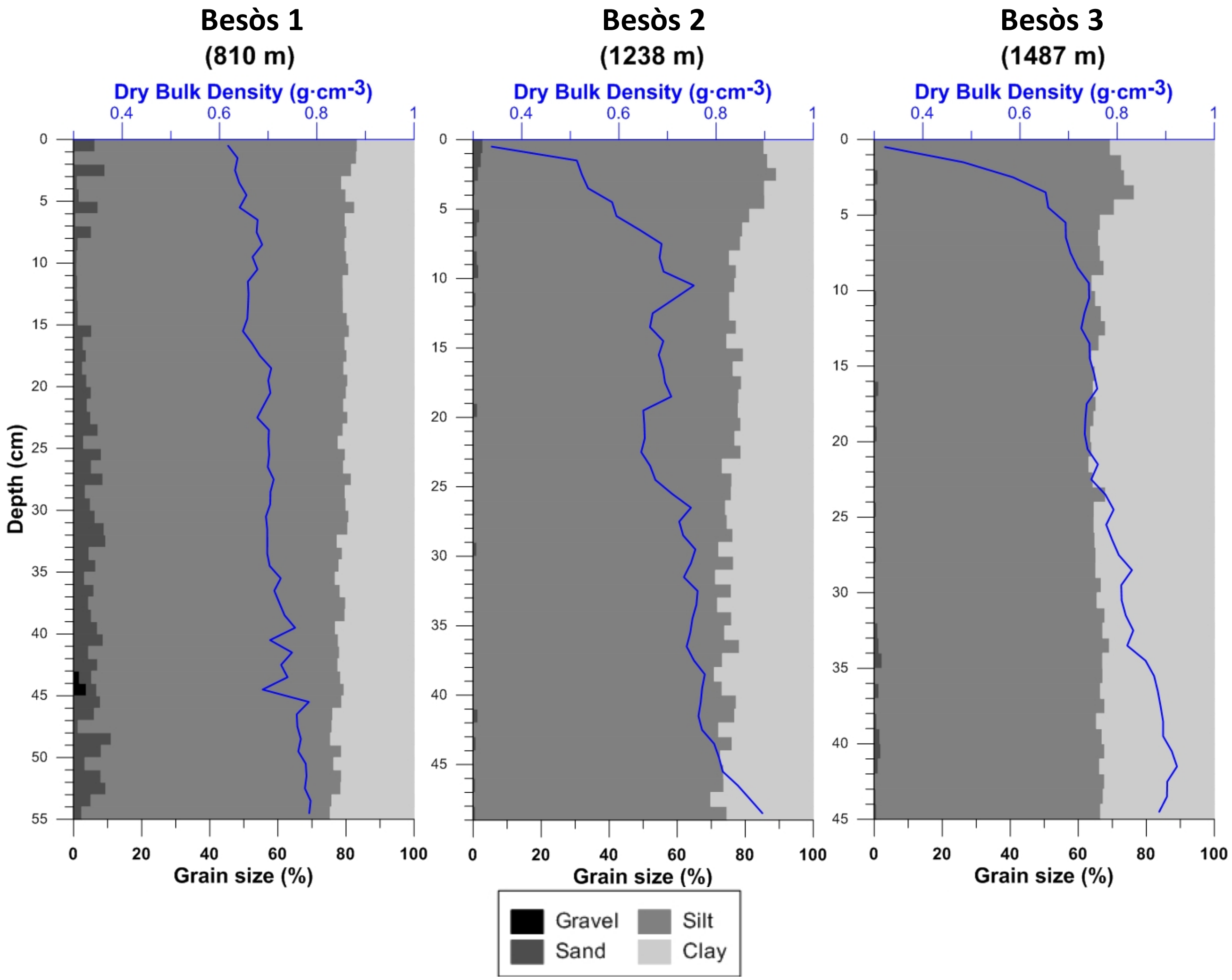

Supplementary Figure S2. Dry bulk density (blue lines) and grain size fraction (grey scales) profiles of sediment cores retrieved in the Besòs Canyon axis.

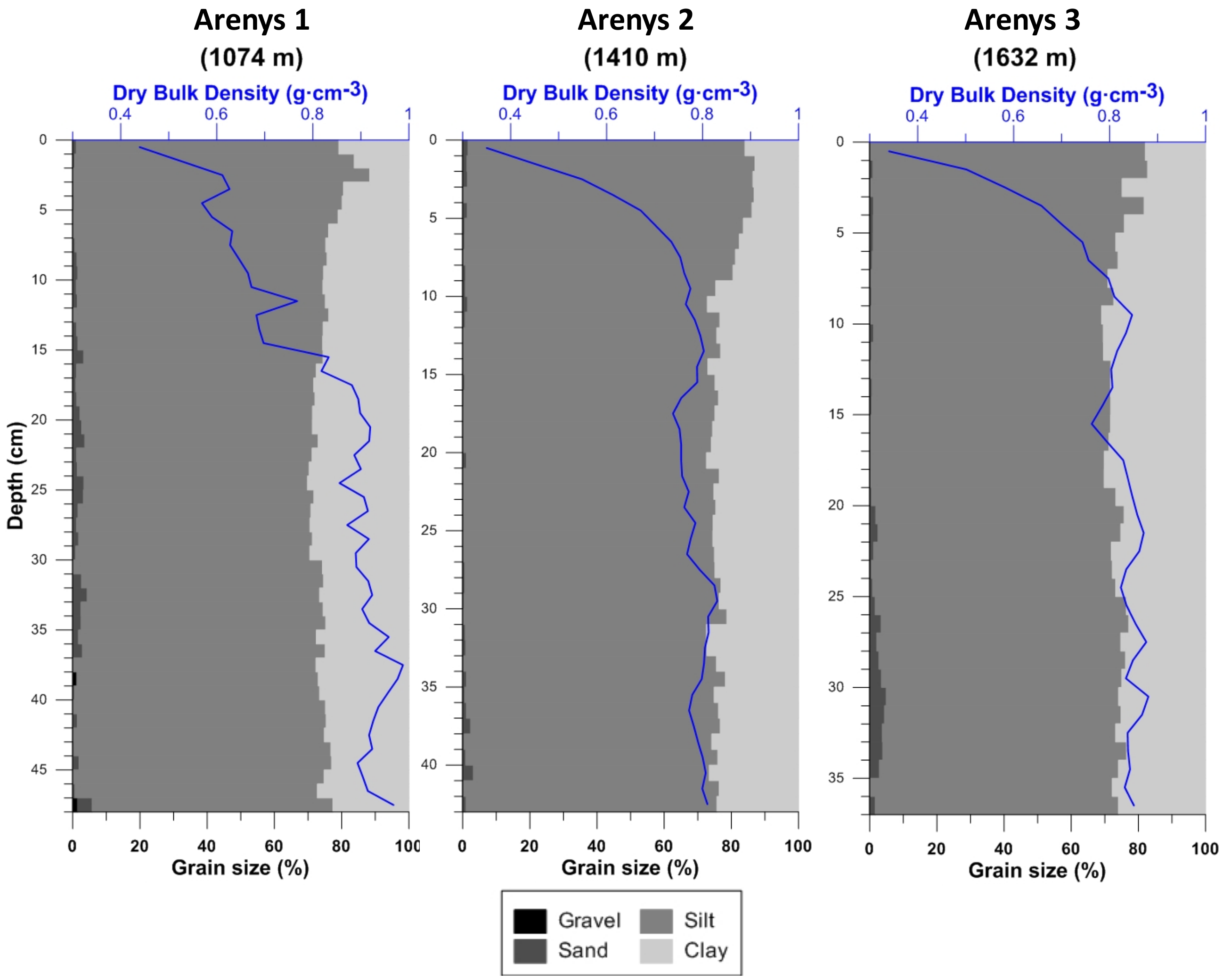

Supplementary Figure S3. Dry bulk density (blue lines) and grain size fraction (grey scales) profiles of sediment cores retrieved in the Arenys Canyon axis.

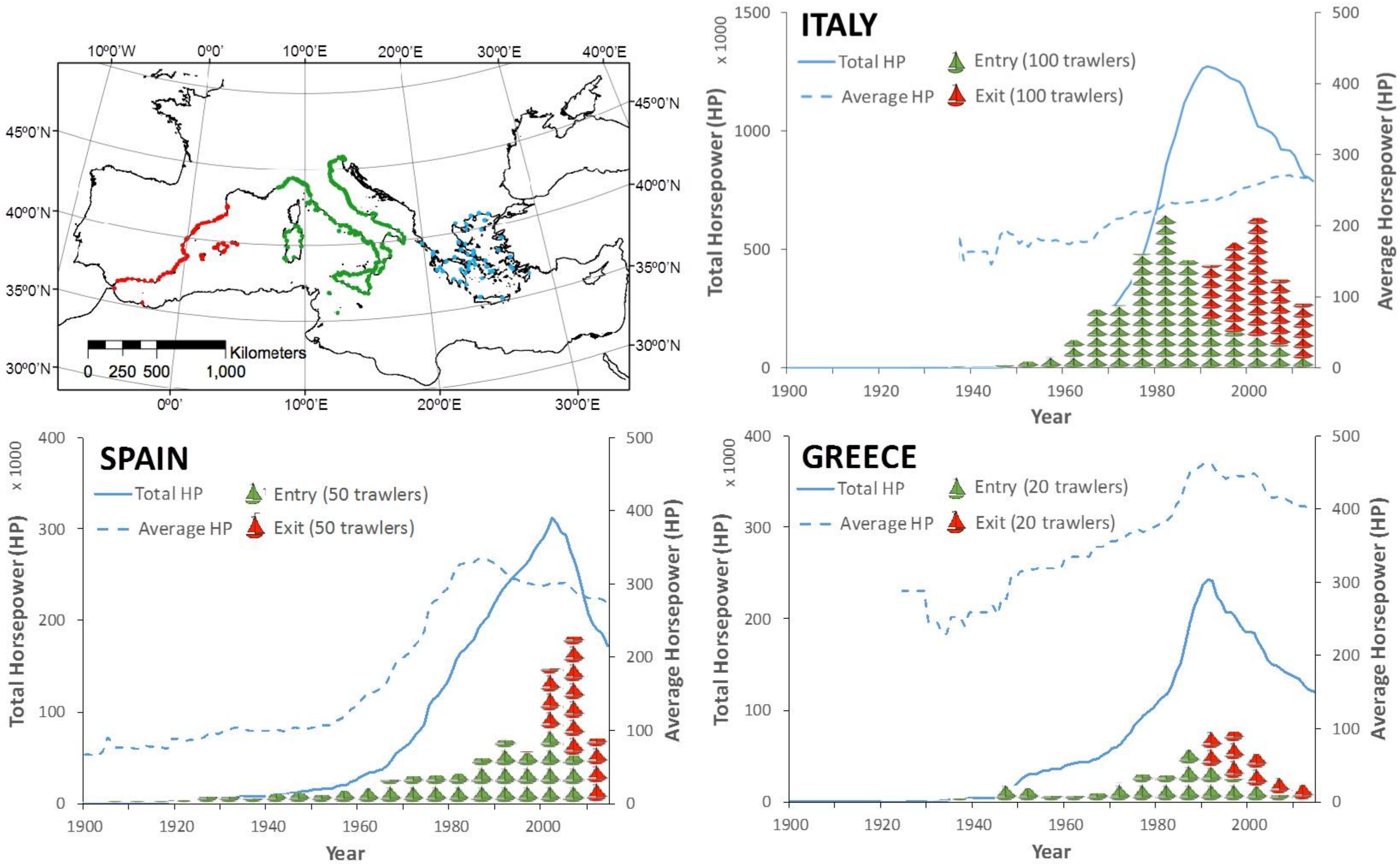

Supplementary Figure S4. Distribution of bottom-trawling ports of the most relevant fisheries in the Mediterranean Sea: Italy, Greece, and Spain. Graphs indicate evolution of each countries' trawling fleets in terms of total horsepower, average horsepower, and entry and removal of vessels. Number of bottom trawlers entering and exiting the active fleet are shown over a five-year interval. Note the greater scales of total horsepower and number of entering and exiting trawlers of Italy. Map was generated using ArcGIS 10.3 (http://desktop.arcgis.com/en/arcmap/10.3/main/get-started/whats-new-in-arcgis.htm).
